# DNA-incorporated thioguanine to detect potential non-adherence to maintenance therapy in acute lymphoblastic leukemia

**DOI:** 10.1007/s00280-025-04784-7

**Published:** 2025-07-16

**Authors:** Mathilde Rønne Koch, Anna Sofie Buhl Rasmussen, Bodil Als-Nielsen, Ximo Duarte, Gabriele Escherich, Mats Heyman, Kristi Lepik, Johan Malmros, Jacob Nersting, Inga Johannsdottir, Riitta Niinimäki, Malene Johanne Petersen, Heidi Segers, Inge Margriet van der Sluis, Maria Thastrup, Goda Vaitkeviciene, Kjeld Schmiegelow, Linea Natalie Toksvang

**Affiliations:** 1 Department of Pediatrics and Adolescent Medicine, University Hospital Rigshospitalet, Copenhagen, Denmark; 2 Departamento de Pediatria, Instituto Português de Oncologia Lisboa Francisco Gentil, Lisbon, Portugal; 3 University Medical Center Hamburg-Eppendorf, Hamburg, Germany; 4 Department of Women’s and Children’s Health, Astrid Lindgren Children’s Hospital, Karolinska University Hospital, Karolinska Institutet, Stockholm, Sweden; 5 Tallinn Children’s Hospital, Tallinn, Estonia; 6 Dept of Ped hematology and oncology, Oslo University Hospital, Oslo, Norway; 7 Department of pediatrics, Oulu University Hospital and Research Unit of Clinical medicine, University of Oulu, Oulu, Finland; 8 Department of Pediatric Hemato-Oncology, University Hospitals Leuven, Leuven, Belgium; 9 Department of Pediatric Oncology, Catholic University Leuven, Leuven, Belgium; 10 Princess Máxima Center for Pediatric Oncology, Utrecht, Netherlands; 11 Center for Pediatric Oncology and Hematology, Vilnius University, Vilnius, Lithuania; 12 Institute of Clinical Medicine, The Faculty of Medicine, University of Copenhagen, Copenhagen, Denmark

**Keywords:** Acute lymphoblastic leukemia, Maintenance therapy, Mercaptopurine, DNA thioguanine, Adherence, Therapeutic drug monitoring

## Abstract

**Purpose:**

Adherence to 6-mercaptopurine (6-MP)/methotrexate maintenance treatment for acute lymphoblastic leukemia (ALL) is pivotal to preventing relapse, and the 6-MP metabolite DNA-incorporated thioguanine (DNA-TG) is associated with relapse risk. In the ALLTogether-1 (A2G1) Maintenance sub-study (EU CT nr 2022-501050-11-01), DNA-TG, thioguanine nucleotides (TGN), and methylated mercaptopurine metabolites (MeMP) are analyzed regularly. Upon levels below preset limits (TGN < 50, or MeMP < 200 or < 100 nmol/mmol hemoglobin for thiopurine S-methyltransferase (TPMT) wild type and heterozygous patients, respectively), the treating physician is informed of potential non-adherence. We investigated the feasibility of using DNA-TG as the primary flagging of potential non-adherence.

**Methods:**

We analyzed 6-MP metabolites in 3,074 blood samples from 368 children enrolled in the A2G1 Maintenance sub-study.

**Results:**

In 6% of samples, TGN (median 212, 95% range 40–642), MeMP (median 4,959, 95% range 135–23,880) or both were below the flagging potential non-adherence limits. DNA-TG was associated with TGN (estimate = 1.72, *p* < 0.0001), MeMP (estimate = 1.10, *p* < 0.0001), and prescribed 6-MP dose (estimate = 1.083 and 1.132, *p* < 0.0001, for TPMT wild type and heterozygous patients) in linear effects models, and the predicted probability of treatment interruption in logistic regression models. DNA-TG was below 200 fmol TG/µg DNA (13th percentile of all measurements, median 569, 95% range 73–1,823) in all samples with both TGN and MeMP below the flagging potential non-adherence limits.

**Conclusion:**

DNA-TG can provide a cost-effective guidance on when to measure TGN and MeMP to determine whether non-adherence should be suspected, which is an additional benefit to monitoring DNA-TG during maintenance therapy.

**Supplementary Information:**

The online version contains supplementary material available at 10.1007/s00280-025-04784-7.

## Introduction

With contemporary treatment, above 90% of children and 70% of young adults with acute lymphoblastic leukemia (ALL) are cured [1, 2], but 10% of patients subsequently develop leukemic relapse [2], resulting in exposure to very toxic 2nd line therapy as well as reduced survival chances [3]. Maintenance therapy with daily 6-mercaptopurine (6-MP) and weekly methotrexate (MTX) is essential to avoid leukemic relapse [4], and the end-point 6-MP metabolite DNA-incorporated thioguanine (DNA-TG) is associated with relapse risk [5, 6]. The primary mode of action for 6-MP is intracellular formation of thioguanine-nucleotides (TGN), which are incorporated into DNA in competition with natural guanine. Random S-methylation of DNA-TG may cause nucleotide mismatching and futile mismatch repair (due to persisting mismatching) and ultimately apoptosis [4]. A competing metabolic pathway for 6-MP and its metabolites is methylation by thiopurine S-methyltransferase (TPMT), creating methylated mercaptopurine metabolites (MeMP), some of which are inactive, while some inhibit de novo purine synthesis, thereby enhancing incorporation of TGN into DNA by decreasing the pool of natural guanine [4] (Online Resource 1). Loss of function TPMT variants decrease enzyme activity, and TPMT heterozygous patients (5–10% of Caucasians) have lower MeMP and higher TGN and DNA-TG, than homozygous wild type patients (90%), but similar relapse rates [7]. About one in 300 is TPMT deficient, requiring significant preemptive dose reduction to avoid life-threatening myelosuppression [4, 7]. Incorporation of TGN into DNA is counteracted by nudix hydrolase 15 (NUDT15) mediated hydrolysis of thio-nucleoside phosphates, and a poor metabolizer genotype is predominately seen in patients of East Asian ancestry (one in 50) [4].

Systemic exposure to 6-MP reflects pharmacogenetics, prescribed drug doses, and patient/caregivers’ adherence to therapy. Adherence with intake of less than 90–95% of the prescribed doses has been associated with a 2.5–3.9-fold increased risk of relapse [9, 10]. Adherence rates have been shown to decrease over the course of maintenance therapy [10–12], and multiple studies report overall adherence rates below 95% [13], with the lowest adherence among adolescents and young adults [14, 15]. Proxy measures for adherence include self- or parents’ report [16], and the use of medication refill records or a micro-electronic chip in the bottle-cap. Pharmacological monitoring, through 6-MP metabolite analyses, provides a more direct measure of 6-MP intake and relevant cytotoxic exposures, although complicated by individual drug disposition, and is thus far based on erythrocyte levels of TGN and MeMP, while the feasibility of DNA-TG to detect non-adherence has not yet been investigated. In studies analyzing both TGN and MeMP, non-adherence is mainly associated with low levels of both metabolites [17, 18], while varying adherence has been associated with high intraindividual variance in TGN [9]. Systematic pharmacological monitoring of 6-MP metabolites is currently implemented as part of the European ALLTogether1 (A2G1) treatment protocol (EU CT nr 2022-501050-11-01), where DNA-TG measurements are performed to confirm the association with relapse, whereas low levels of either MeMP or TGN generate a reporting back notice to the treating physician, who can assess the likelihood of non-adherence. In this study, we evaluated the current system for monitoring of potential non-adherence using prescribed treatment interruptions as a proxy for non-adherence and examined the potential of using DNA-TG in the pharmacological monitoring of adherence to maintenance therapy, to increase cost-efficacy of metabolite monitoring.

## Methods

### Study population

After obtaining written informed consent from the patient and/or legal guardians, study participants were enrolled in the A2G1 Study Protocol and the A2G Maintenance Therapy sub-study, from 36 treatment centers in Belgium, Denmark, Estonia, Finland, Germany, Lithuania, the Netherlands, Norway, Portugal, and Sweden. All patients on the A2G1 protocol receiving maintenance therapy were eligible for the Maintenance Therapy sub-study (full list of A2G1 inclusion/exclusion criteria in Online Resource 2), with the exception of patients who were included in a three arm randomization with standard maintenance therapy either preceded by six weeks of treatment with Inotuzumab Ozogamicin or supplemented with low-dose 6-thioguanine enhanced ALL maintenance (TEAM) [19].

**Fig. 1:**
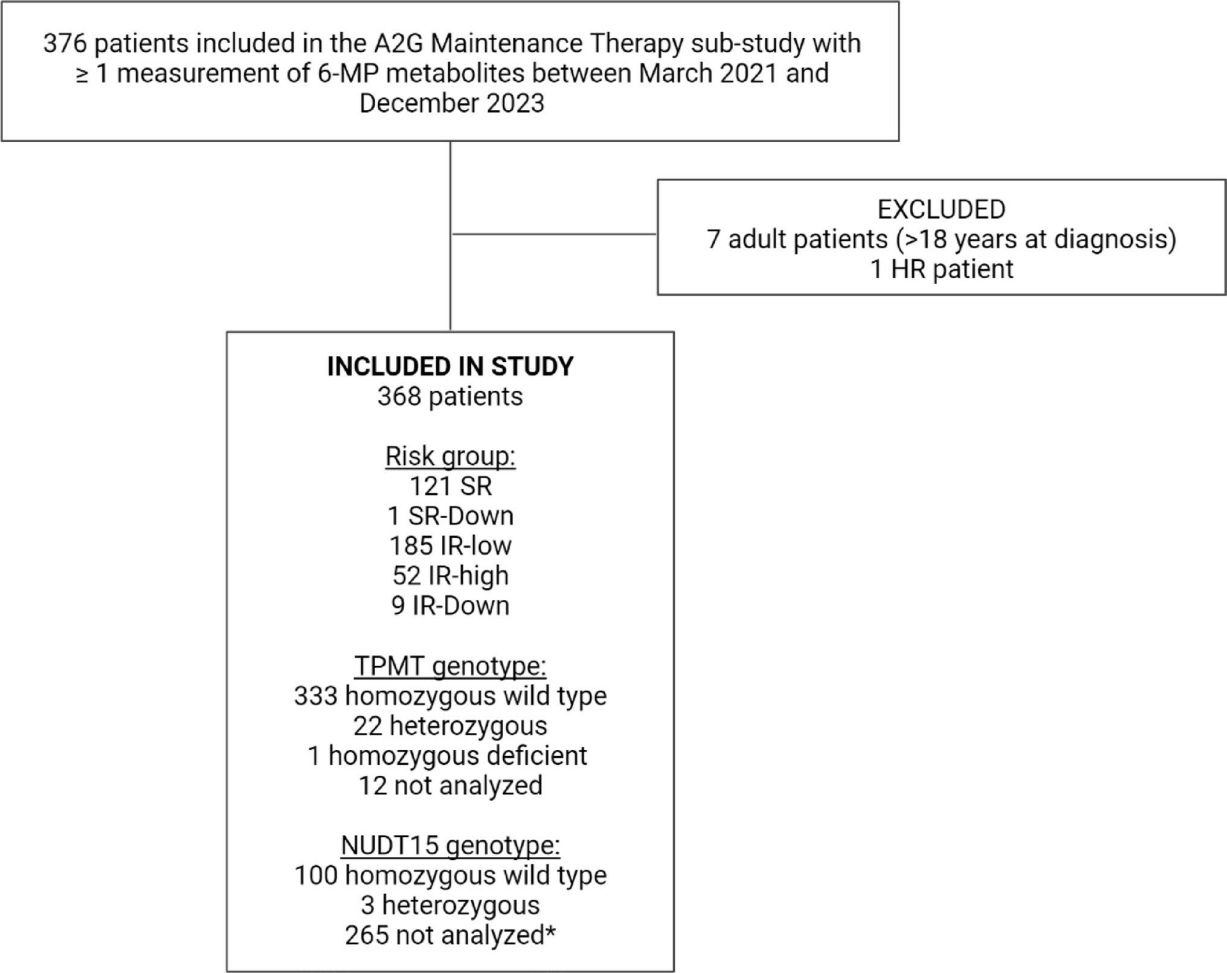
Consort diagram of included patients. A2G: ALLTogether, SR: standard risk, SR-Down: standard risk Down syndrome, IR-low: intermediate risk-low, IR-high: intermediate risk-high, IR-Down: Intermediate risk Down syndrome, HR: high risk, TPMT: thiopurine S-methyltransferase. NUDT15: nudix hydrolase 15. *No indication for the analyses for 251 of these patients, NUDT15 analysis only mandatory for patients of Asian ancestry in the A2G1 protocol. Created with BioRender.com

Inclusion criteria for this study were inclusion in the A2G Maintenance Therapy sub-study, age *≤* 18 years, and ≥ one blood sample collected between March 19th 2021 and November 11th 2023. One patient stratified to high risk (HR) was excluded. We included 368 patients (Fig. 1).

### Risk group stratification

Following one month of a three- or four-drug induction therapy stratified according to the National Cancer Institute (NCI) criteria [20], minimal residual disease (MRD) was measured by flowcytometry and PCR at day 29 and day 71. See Online Resource 3 and 4 for flowcharts of the stratification. Patients with high risk (HR) ALL were excluded from the present study.

Patients with B-cell-precursor (BCP) ALL, no high-risk genetics^1^, no ABL-class fusions, and no CNS involvement were stratified to standard risk (SR) if they had undetectable MRD on day 29, and to intermediate risk (IR)-low if they were < 16 years, had no CNS involvement, detectable MRD < 5% on day 29^2^ and MRD < 0.05% on day 71 (if no other conditions for a higher risk group are fulfilled). Patients with T-cell ALL were stratified as IR-low by almost the same criteria, only undetectable MRD at day 71. Patients who did not meet the criteria for SR, IR-low or HR, including MRD or genetic analysis failures, were stratified as IR-high, as well as patients with T-cell ALL aged < 16 years with day 29 MRD ≥ 5% if they had day 50 MRD < 0.5% and undetectable MRD at day 71. Patients with Down syndrome were stratified as standard risk (SR-Down) if the criteria for SR were fulfilled, and no IKZF1-deletion were present, otherwise as intermediate risk (IR-Down) at detectable MRD < 5% at day 29.

### Maintenance therapy

Maintenance therapy was initiated at week 25 of therapy for SR, week 27 for SR-Down, week 32 for IR-low, and week 37 for IR-high and discontinued after a total of 2 years from end of induction. Patients received oral 6-MP in the starting dose of 75 mg/m^2^/day (5 mg/m^2^/day for TMPT deficient or compound heterozygous TPMT and NUDT15 patients) and oral MTX in the starting dose of 20 mg/m^2^/week, which was titrated to a target level of absolute neutrophile count (ANC, 0.75–1.5*10^9^/L) and platelets (> 75*10^9^/L). The dose of 6-MP and MTX should be reduced to 50% upon ANC 0.5–0.75*10^9^/L and/or platelets 50–75*10^9^/L, and interrupted at ANC < 0.5*10^9^/L or platelets < 50*10^9^/L.

Furthermore, all patients received intrathecal therapy in various total numbers with MTX or triples according to age and CNS stratification^3^. Pulses of vincristine 1.5 mg/m^2^ (one dose) and dexamethasone 6 mg/m^2^/day (five days) every four weeks was added for IR-high patients, while IR-low patients were randomized to standard maintenance with or without pulses (part of another A2G sub-study).

### 6-MP metabolites and limits for reporting back

Samples of 1–5 ml of whole blood in an EDTA tube were collected at monthly intervals (mandatory every three months) and sent with regular mail (except for Belgian samples that were sent frozen in batches) to the Pediatric Oncology Research Laboratory in Copenhagen, Denmark, for analysis of 6-MP metabolites. The median time of transport for the samples sent by regular mail was three days (75% range 1–6 days).

DNA-TG was measured in leukocytes and reported as fmol TG/µg DNA. After extraction of ~ 2 µg of DNA, followed by depurination, purine nucleobases were ethenoderivatized with chloroacetaldehyde, and quantified by tandem mass spectrometry [21], using isotope internal standards. TGN and MeMP were measured in whole blood and reported in nmol/mmol hemoglobin. TGN and MeMP were quantified with ultraviolet ultra-performance liquid chromatography with PDA detection (UPLC-PDA). Sample extraction was performed by deproteinization with perchloric acid (in the presence of dithiothreitol (DTT)) followed by acidic hydrolysis at 99⁰C (based on the method described by Dervieux, T. et al. [22], with measures in whole blood instead of erythrocytes). TGN and MeMP in blood samples are affected by the transport time of samples. Stability analyses from the Peadiatric Oncology Research Laboratory, Copenhagen, Denmark show a median reduction of TGN of approx. 8% (SD 2.8%) after two days, 24% (SD 4.2%) after five days and 39% (SD 6.5%) after seven days, and a reduction of MeMP of approx. 5% (SD 7.2%) after two days, 13% (SD 8.4%) after five days and 14% (SD 23.3%) after seven days, though the recovery of MeMP seemed to vary substantially between patients (Online Resource 5). DNA-TG is more stable with no changes in concentration during a 14-day period at room temperature (data not shown). Values of TGN and MeMP were classified as “below the detection limit” if they were below the mean of the lowest standard (< 82 nmol/l for TGN and < 617 nmol/l for MeMP) before division with hemoglobin level, corresponding to < 12 and < 88 nmol/mmol hemoglobin in a typical blood sample with 7 mmol/L hemoglobin. We calculated the mean DNA-TG, MeMP and TGN in case of more than one sample per day.

Based on a large historical dataset from the Nordic Society of Paediatric Haematology and Oncology (NOPHO) ALL-2008 Maintenance study, the limits for generation of a reporting back notice in the A2G Maintenance Therapy sub-study were beforehand defined as TGN < 50 nmol/mmol hemoglobin (< 1 st percentile) *or* MeMP < 200 or 100 nmol/mmol hemoglobin (< 2.5th percentile and < 10th percentile) for TPMT wild type and heterozygous patients, respectively.

### Drug doses

We collected data on prescribed drug doses and the patient’s height and weight along with every blood sample, and calculated drug doses per body surface area. Drug dose was defined as the latest prescribed dose of 6-MP in mg/day preceding the date of sample collection. We screened comments from physicians, enclosed with each sample, for prescribed treatment interruptions or in case of missing data on 6-MP dose. We defined prescribed treatment interruptions as doses of 6-MP = 0 mg/day, or if a comment indicated a current physician-prescribed interruption of treatment with 6-MP, starting at least one day preceding sample collection. For patients from Rigshospitalet Copenhagen in Denmark, data on drug doses was obtained directly from the treatment records. Data on dose of 6-MP in mg/day was available for 2,532 metabolite samples, and dose of 6-MP in mg/day/m^2^ was available for 2,207 metabolite samples.

### Statistical analyses

Statistical analyses, plots and tables were made using R, version 4.3.0 [23–25]. All statistical analyses were performed on complete datasets, as we excluded data with missing values on any of the variables considered in the specific analysis. All associations are presented as estimates with nominal 95% confidence intervals; no p-values have been adjusted for multiple tests.

The median DNA-TG, MeMP and TGN was calculated for each separate patient (_m_DNA-TG, _m_MeMP, _m_TGN), and the Mann Whitney U test was used to compare median _m_DNA-TG, _m_TGN, and _m_MeMP across patient groups. All pairwise associations between variables were visualized using locally estimated scatterplot smoothing (LOESS). Associations of DNA-TG with MeMP, TGN and 6-MP dose were assessed on logarithmically transformed data, using linear mixed effects models (R-function *lmer)*. Associations between 6-MP metabolites and probability of a prescribed treatment interruption were assessed using mixed effects logistic regression models (R function *glmer*) and reported as the odds ratio (OR) for treatment interruption. All associations were assessed in mutually adjusted models, a model additionally adjusted for transport time of the samples, and a model further adjusted for age and sex, and the association 6-MP metabolites with probability of a prescribed treatment interruption was further assessed when excluding samples with more than two transport days. Furthermore, all statistical analyses included a random intercept of subject and treatment center to account for the correlation between observations on the same patient or on patients from the same treatment center.

Sensitivity and specificity of the logistic regression models were compared graphically by receiver-operating-characteristic (ROC) curves, and the area under the curve (AUC) and selected partial areas under the curve (pAUC) were calculated for each ROC curve. (see Online Resource 6 for a detailed description of statistical analyses).

## Results

### Patient population and metabolite samples

In total, 368 patients were included in this study (see Online Resource 7 for number of patients and samples from each country). Median age at diagnosis was 4.7 years (min 0.4, max 17.2), 47% were female and 53% were male. Most patients presented with BCP ALL (93.2%), were TMPT homozygous wild type (93.5%) and NUDT15 homozygous wild type (97.1%, not analyzed for 265 patients). One patient was compound TPMT and NUDT15 heterozygous. The patients were stratified as IR-low (*n* = 185, 50.3%), SR (*n* = 121, 32.9%), IR-high (*n* = 52, 14.1%), SR-Down (*n* = 1, 0.3%) and IR-Down (*n* = 9, 2.4%) (Online Resource 8). Median time between sample collection for individual patients was 29 days (75% range 27–41, max 209). The median number of samples collected per patient was seven (min one, max 34), while 36 patients (9.8%) contributed with only one blood sample.

### Samples detected with the current reporting back system

Of 3,021 samples with measures of both TGN or MeMP, 187 samples (6.2%) from 96 patients had either TGN or MeMP below the reporting back limits. Of these, 39 samples from 26 patients had levels of *both* TGN and MeMP below the limits, corresponding to 1.3% of the total samples.

Of the 26 patients with a sample with both TGN and MeMP below the reporting back limit, 17 (65%) were male and their median age was 5.0 years (min 1.4, max 16.7). Of these patients, 21 had only one sample with both metabolites below the reporting back limits, while five had two or more (maximum seven from the same patient). The latter five patients had a total of 51 samples available, with 35% having low levels of both TGN and MeMP. The samples with both low MeMP and TGN were distributed throughout maintenance therapy, but six were collected on the first day of maintenance therapy (three IR-high, two IR-low, one SR), thereby not reflecting non-adherence (Online Resource 9). Five of the 39 samples with low TGN and MeMP were collected during a registered prescribed treatment interruption, while 20 of these samples were not collected during a treatment interruption (missing information on treatment interruption status for 14 samples).

### Distribution of 6-MP metabolites

**Fig. 2:**
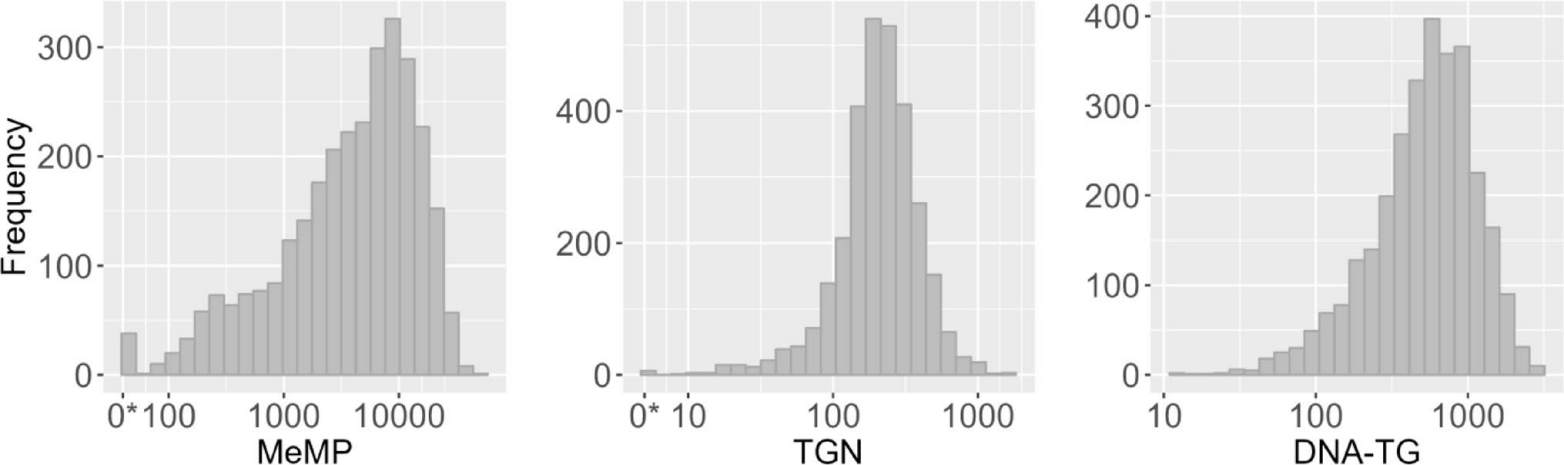
Distribution of methylated mercaptopurine metabolites (MeMP), thioguanine nucleotides (TGN) and DNA incorporated thioguanine nucleotides (DNA-TG) in the collected blood samples. *Below the detection limit

A total of 3,074 samples from 368 patients were included in the study, whereof 2,990 samples from 364 patients had measurements of all three metabolites, MeMP, TGN and DNA-TG. Distributions of MeMP, TGN and DNA-TG were right-skewed; log-transformed values of TGN and DNA-TG improved symmetry (Fig. 2). MeMP had a wider range (median 4,959; 95% range 135–23,880; min below the detection limit; max 45,306) than TGN (median 212; 95% range 40–642; min below the detection limit; max 1,529) and DNA-TG (median 569; 95% range 73–1,823; min 13; max 3,043). The limits for generation of a reporting back notice in the A2G Maintenance Therapy sub-study corresponded to the 3.6th percentile for TGN (< 50) and the 3.7th and 5.9th percentile for MeMP (< 200 for TPMT wild type and < 100 for TPMT heterozygous).

The total median of _m_DNA-TG (median DNA-TG for each patient) was 585 fmol TG/µg DNA, total median _m_MeMP was 4,712 nmol/mmol hemoglobin and total median _m_TGN was 217 nmol/mmol hemoglobin. The median _m_DNA-TG was higher in males than females (638 vs. 512 nmol/mmol hemoglobin, *p* = 0.003), furthermore, the _m_DNA-TG was somewhat higher in SR patients compared to IR-low patients (659 vs. 541, *p* = 0.005) and IR-high patients (659 vs. 559, *p* = 0.047), while there was no significant difference between the two IR groups (*p* = 0.85). The median _m_TGN and _m_MeMP were comparable across sexes and risk groups, and no significant differences were found across phenotypes (BCP and T-cell ALL) for both _m_DNA-TG, _m_TGN, and _m_MeMP (Online Resource 10). The total median DNA-TG increased throughout maintenance, as seen by a positive slope of the LOESS line of the median DNA-TG and days in maintenance therapy, which was not seen for MeMP and TGN (Online Resource 11).

### Associations of DNA-TG with MeMP, TGN and prescribed dose of 6-MP

**Fig. 3:**
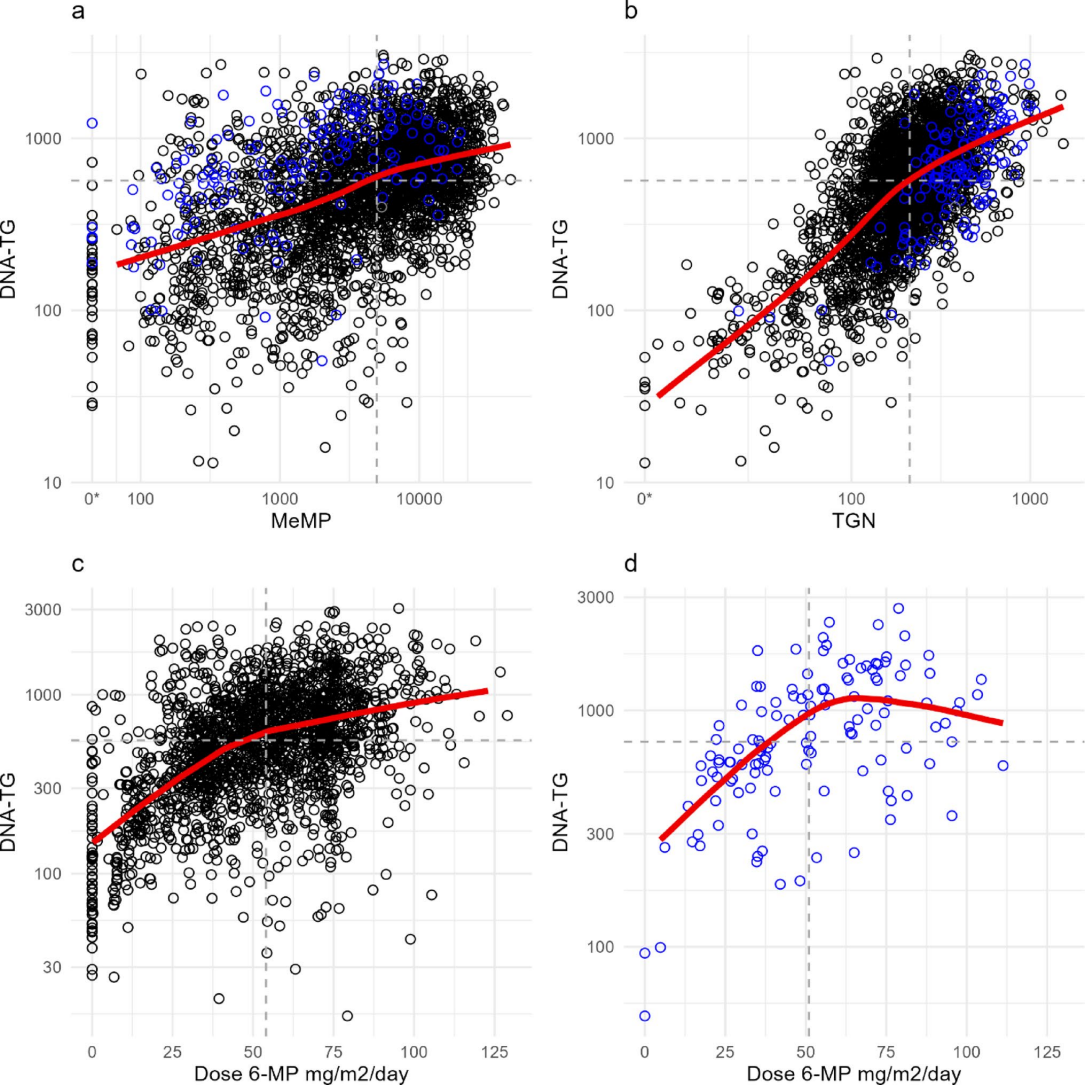
Plots of measured 6-mercaptopurine (6-MP) metabolites and prescribed doses of 6-MP with locally estimated scatterplot smoothing (LOESS, red line). Samples from one thiopurine S-methyltransferase (TPMT) deficient patient not included. Black points: samples from TPMT homozygous wild type patients, blue points: samples from TPMT heterozygous patients. Dotted line: median value. **a** Mercaptopurine metabolites (MeMP) and DNA incorporated thioguanine nucleotides (DNA-TG, 2,991 samples from 363 patients), **b** thioguanine nucleotides (TGN) and DNA-TG (2,992 samples from 363 patients), **c** dose of 6-MP in mg/m^2^/day and DNA-TG for TPMT homozygous wild type patients (2,066 samples from 317 patients, 17 samples with dose of 6-MP > 130 mg/m^2^/day not shown), **d** dose of 6-MP in mg/m^2^/day and DNA-TG for TPMT heterozygous patients (128 samples from 21 patients). *Value below the detection limit

Visually, there was a positive association between TGN and MeMP, however, some of the highest levels of TGN were seen with the lowest levels of MeMP, with a LOESS line with MeMP as outcome variable flattening due to the high TGN levels at moderate and low levels of MeMP (Online Resource 12). A positive association of DNA-TG with both MeMP and TGN was observed (Fig. 3a and b), with a steeper LOESS line for association with TGN than MeMP. Furthermore, a positive association of DNA-TG with prescribed 6-MP dose in mg/m^2^/day was observed, though the LOESS line flattened and reversed at higher 6-MP doses for TPMT heterozygous patients (Fig. 3c and d).

In linear mixed effects models, DNA-TG was positively associated with TGN and MeMP in mutually adjusted analyses. DNA-TG increased by 10% at a doubling of MeMP (estimate 1.10, 95% CI 1.09–1.12) and increased by 72% at a doubling of TGN (estimate 1.72, 95% CI 1.68–1.77), at fixed effects of the other metabolite. There was a positive association of DNA-TG with 6-MP dose for both TPMT genotypes, with an increase in DNA-TG of 8% (estimate 1.08, 95% CI 1.07–1.09) and 13% (estimate 1.13, 95% CI 1.09–1.18) at a 20% increase of 6-MP dose for the TPMT homozygous wild type and heterozygous patients, respectively. Similar estimates for association of DNA-TG with MeMP, TGN and dose of 6-MP were seen after adjusting for transport days of the metabolite samples and additionally for age and sex (Online Resource 13 and 14).

### DNA-TG in samples with low TGN and MeMP

**Fig. 4:**
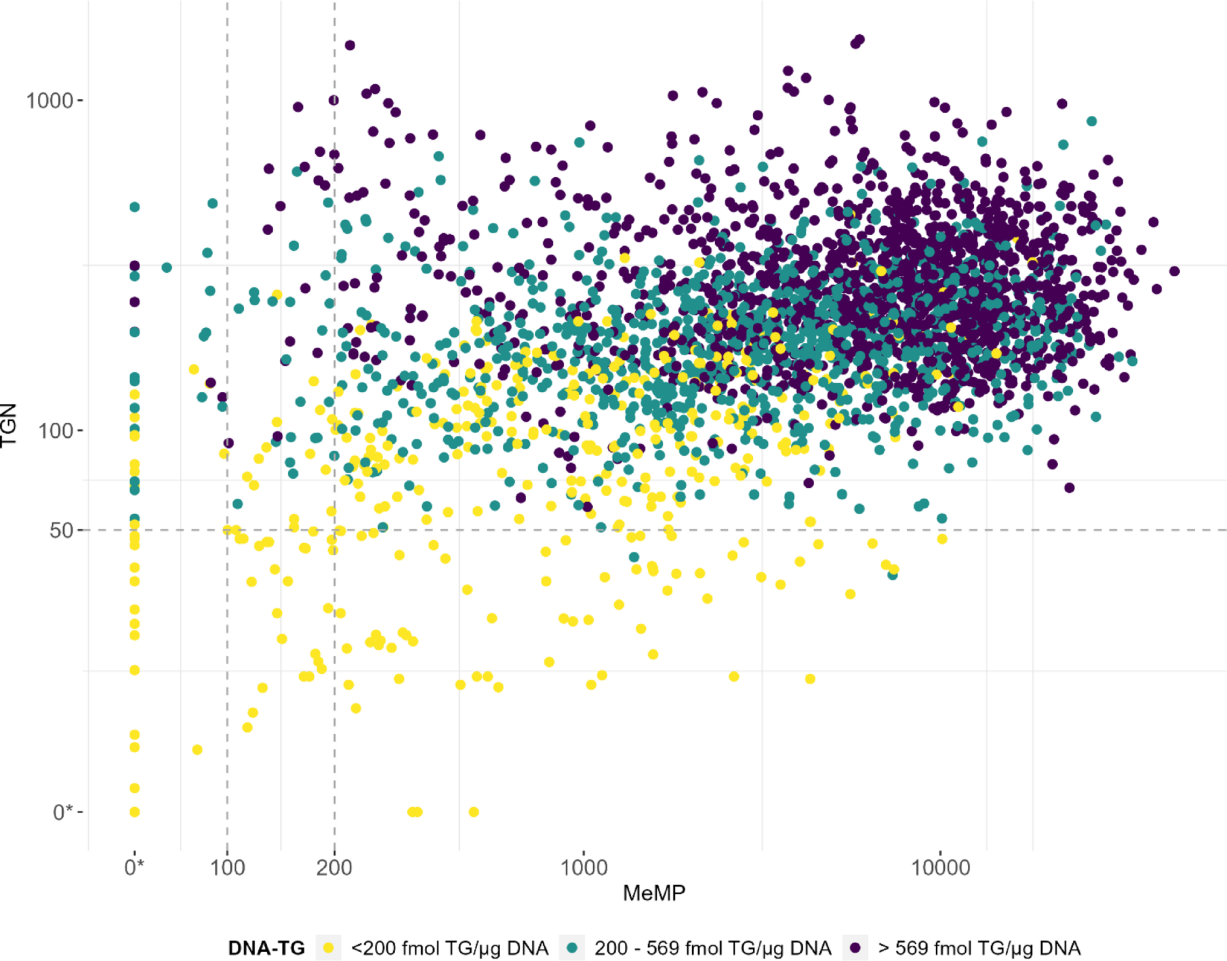
Plot of methylated mercaptopurine metabolites (MeMP) and thioguanine nucleotides (TGN) in 2,990 blood samples from 364 patients. The corresponding measurement of DNA incorporated thioguanine nucleotides (DNA-TG) in the sample is illustrated by color. Median DNA-TG = 569 fmol TG/µg DNA. The limits for generation of a reporting back notice for MeMP and TGN are illustrated as dotted lines (MeMP limit dependent on the TPMT genotype). *Below detection limit

DNA-TG was correspondingly low in samples with both MeMP and TGN below the reporting back limit in the same blood sample (Fig. 4); the maximum value of DNA-TG in these samples was 198 fmol TG/µg DNA. Of all 3.074 samples, 399 had DNA-TG below 200 fmol TG/µg DNA, which corresponds to the 13th percentile, and of these samples with low DNA-TG approx. 10% had TGN and MeMP below the reporting back limits. DNA-TG was predominantly below 200 fmol TG/µg DNA in samples with TGN below the reporting back limit at all levels of MeMP, while higher values of DNA-TG is seen in samples with low MeMP and high TGN, with the maximum of 2,367 fmol TG/µg DNA (above the 99th percentile) in samples with only MeMP below the reporting back limit.

### Probability of treatment interruption

Logistic regression analyses were performed on 2,455 samples from 349 patients with complete data on 6-MP metabolites and 6-MP dose in mg/day and showed an association between levels of 6-MP metabolites and the probability of a prescribed treatment interruption.

In univariate models, decreasing levels of DNA-TG, TGN and MeMP were associated with a prescribed treatment interruption (Table 1). In a mutually adjusted model with all metabolites, the predicted probability of a prescribed treatment interruption decreased 59% per doubling of DNA-TG (OR 0.41, 95% CI 0.29–0.58) and 87% per 100 nmol/mmol hemoglobin increase in TGN (OR 0.13, 95% CI 0.06–0.26), with similar estimates when additionally adjusting for age, sex, and transport time of the samples, as well as when excluding samples with more than two days of transport (Online Resource 15). In univariate models, the probability of a prescribed treatment interruption increased with decreasing MeMP, but the association was reversed when mutually adjusting for either DNA-TG, TGN or both, possibly due to reverse causation, leading to an exclusion of MeMP in succeeding analyses.

**Table 1:** Associations of DNA incorporated thioguanine nucleotides (DNA-TG), methylated metabolites (MeMP) and thioguanine nucleotides (TGN) with the predicted probability of a prescribed treatment interruption, in general linear mixed effects models with a random effect of patients and treatment center (analyses performed on 2,455 6-MP metabolite measurements from 349 patients with complete data on metabolites and status of prescribed treatment interruption at the time of sample collection)

	Univariate model			Mutually adjusted model			Mutually adjusted with TGN and DNA-TG		
	OR	95% CI	p-value	OR	95% CI	p-value	OR	95% CI	p-value
DNA-TG per doubling	0.24	0.18–0.31	< 0.0001	0.41	0.29–0.58	< 0.0001	0.46	0.33–0.63	< 0.0001
TGN per 100 nmol/mmol hemoglobin	0.06	0.03–0.11	< 0.0001	0.13	0.06–0.26	< 0.0001	0.17	0.09–0.32	< 0.0001
MeMP per doubling	0.64	0.55–0.74	< 0.0001	1.34	1.07–1.68	0.0105			

**Fig. 5:**
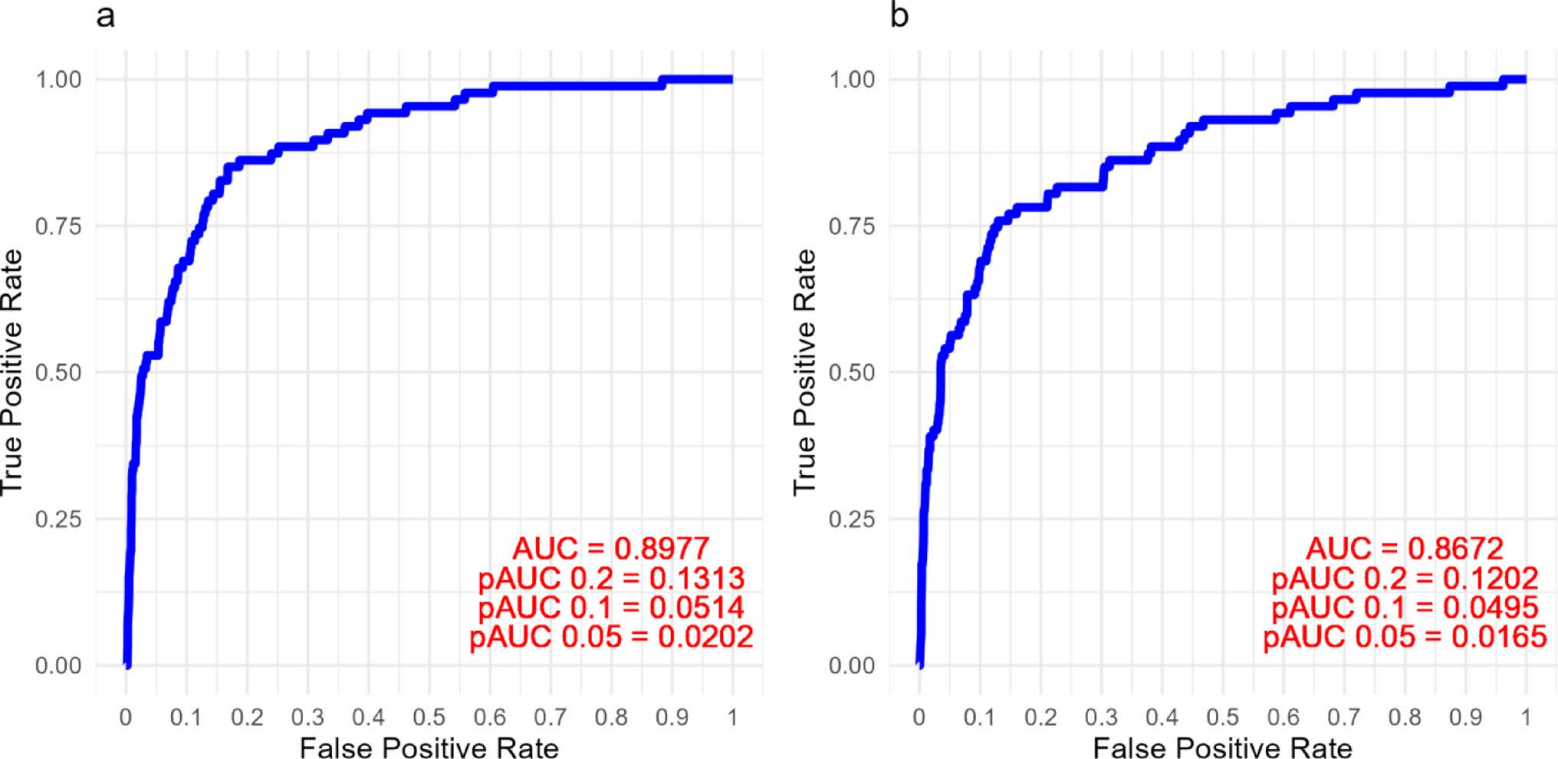
Receiver-operating-characteristic (ROC) curves, illustrating the performance of general mixed effect models to predict the probability of a prescribed treatment interruption at the time of sample collection, based on 6-mercaptopurine (6-MP) metabolite levels. **a** Model with thioguanine nucleotides (TGN) and DNA incorporated thioguanine nucleotides (DNA-TG) as predictors, **b** Model with only DNA-TG as predictor. AUC = area under the curve, pAUC = partial area under the curve at given False Positive Rate

ROC-curves were used to illustrate the performance of the models, and the mutually adjusted model with TGN and DNA-TG yielded an AUC of 0.90 and a pAUC_0.1_ (at False Positive Rate = 0.1) of 0.05. The performance of the model was slightly reduced when only using DNA-TG as a predictor of probability of a prescribed treatment interruption (univariate model), with an AUC of 0.87, while almost similar at lower levels of the False Positive Rate (pAUC_0.1_ at 0.05) (Fig. 5).

## Discussion

Monitoring DNA-TG during maintenance therapy is relevant since lower levels has been associated with increased risk of relapse [5, 6]. The findings of this study support the additional use of DNA-TG in the monitoring of adherence to ALL maintenance therapy, as all samples with both low TGN and MeMP had correspondingly low DNA-TG. The results are further supported by the positive associations of DNA-TG with dose of 6-MP, as well as models predicting prescribed treatment interruptions, as a proxy for non-adherence, which performed almost equally well when only basing predicted probabilities on DNA-TG. However, the reverse causation of MeMP with prescribed treatment interruptions problematizes the use of prescribed treatment interruptions as a proxy for non-adherence.

DNA-TG have been shown to integrate upstream 6-MP and MTX metabolites [5, 26]. Total DNA-TG measured in leukocytes represents DNA-TG in polymorph nucleated granulocytes (mainly neutrophils) and mononucleated cells (mainly leukocytes). Total DNA-TG in blood primarily reflects the levels in neutrophils as they generally have higher DNA-TG levels [27]. Since the lifespan of neutrophils is only a few days, total DNA-TG will drop rapidly in case of treatment interruption. However, DNA-TG will not reach zero due to its persistence in long-lived mononuclear cells [27]. Further supporting the feasibility of DNA-TG for adherence monitoring, the metabolite does not decay after sampling, and samples can therefore be sent with regular mail from local treatment centers. DNA-TG samples are usually analyzed within 14 days of receipt at the laboratory.

DNA-TG is associated with risk of relapse [5, 6], whereas this has not been demonstrated for TGN and MeMP in newer studies [5, 28]. Thus, DNA-TG is a promising marker for therapeutic drug monitoring and is monitored throughout maintenance therapy in A2G1 to confirm this association. Since all samples with both TGN and MeMP below the preset limits of reporting back had DNA-TG < 200 fmol TG/µg DNA, this level of DNA-TG can be used to guide which samples to further analyze for MeMP and TGN. Reporting back on all samples with DNA-TG < 200 fmol TG/µg DNA would result in overreporting of suspected non-adherence, since 13% of all samples had DNA-TG below this limit, necessitating additional measurements of TGN and MeMP in samples with low DNA-TG to distinguish potential non-adherence from a skewed metabolism, as high TGN or MeMP in these samples will contradict non-adherence. The current price of a DNA-TG analysis in a blood sample is ~ 124 euro, and ~ 83 euro for an analysis of TGN and MeMP. Only analyzing TGN and MeMP in samples with DNA-TG < 200 fmol TG/µg would decrease these analyses with approximately 90%, thereby greatly increasing cost effectiveness of the system, without (or only rarely) missing patients with TGN and MeMP below the reporting back limits. The investigated system for detection of potential non-adherence generated a notice to the treating physician for 6.2% of the samples but is expected to overestimate non-adherence by addressing TGN and MeMP independently while also not accounting for prescribed treatment interruptions. The strategy for reporting back should be reconsidered, in the interest of increasing the specificity and reducing unnecessary notices to physicians; only reporting back on samples with both low MeMP and TGN would decrease the notices to 1.3% of the samples, thus significantly increasing the signal to noise ratio. However, keeping in mind that the true frequency of non-adherence or inconsistent adherence in this cohort is not known. Moreover, not reporting back on samples collected during prescribed treatment interruption would further reduce notices but requires real-time registration of drug doses. Another possibility to increase sensitivity to inconsistent adherence, resulting in high variation of metabolites but with measurements above the reporting back limits, is to implement intra-patient variance in the system, but this also requires real-time data modeling.

DNA-TG has been suggested as a target for therapeutic drug monitoring during maintenance therapy [5]. Future clinical trials replacing current hematological titration strategies with DNA-TG should include further evaluation of pharmacological adherence monitoring using DNA-TG.

Limitations of this study include no behavioral measure of adherence to confirm suspected non-adherence in cases with low metabolites. Furthermore, the distribution of risk groups in this study is not representative, since 49 IR-high patients participating in the A2G protocol randomization implicating maintenance therapy were not eligible for the present study. The number of patients included, as well as multiple metabolite samples collected for most patients, is adding to the strength of this study. Furthermore, all metabolite samples were analyzed by the same laboratory, increasing comparability between measurements.

## Conclusion

This study supports the use of DNA-TG in pharmacological monitoring of non-adherence during ALL maintenance, which has not yet been explored. Deciding which samples to further analyze for TGN and MeMP based on DNA-TG measurements greatly increases cost effectiveness, potentially increasing implementation of systematic pharmacological monitoring in the clinic. This analytical strategy has now been implemented in the European A2G1 study.

## Electronic supplementary material

Below is the link to the electronic supplementary material.

Supplementary Material 1

## Data Availability

The data that support the findings of this study are available on reasonable request from the corresponding author LNT. The data is not publicly available due to them containing information that could compromise research participant privacy.

## Footnotes

1 High risk genetics: KMT2A/MLL gene fusions, near haploidy, low hypodiploidy (< 40 chromosomes), iAMP21, t(17;19)/TCF3-HLF.

2 MRD at day 29 < 0.1% if t(12;21)/*ETV6-RUNX1* positive or < 0.03% at day if high hyperdiploid or < 0.05% if B-other and a good risk copy number alteration profile (CNA-profile).

3 SR and IR-low every 12th week for a total of three times, SR-Down every 12th week for a total of seven times, IR-high every 12th week for a total of six times, IR-Down and HR-Down every 12th week throughout maintenance and HR every 8th week throughout maintenance.
