# Supplementary material for: DNA-incorporated thioguanine to detect potential non-adherence to maintenance therapy in acute lymphoblastic leukemia
Source: Cancer Chemother Pharmacol. 2025 Jul 16;95(1):76. doi: 10.1007/s00280-025-04784-7 (PMC12267380; doi:10.1007/s00280-025-04784-7)
Supplement: Supplementary file 1 — Supplementary Material 1 [file 280_2025_4784_MOESM1_ESM.docx]

Supplemental material

**Title:** DNA-incorporated thioguanine to detect potential non-adherence to maintenance therapy in acute lymphoblastic leukemia

**Journal name**: Cancer Chemotherapy and Pharmacology

**Names of the authors and affiliation:**

Mathilde Rønne Koch^1^. Anna Sofie Buhl Rasmussen^1^ (ORCID  0000-0002-4997-2914). Bodil Als-Nielsen^1^. Joaquin Duarte^2^ (ORCID  0000-0001-5332-0284). Gabriele Escherich^3^. Mats Heyman^4^. Kristi Lepik^5^. Johan Malmros^4^. Jacob Nersting^1^ (ORCID [0000-0001-9144-7972](https://orcid.org/0000-0001-9144-7972)). Inga Johannsdottir^6^. Riitta Niinimäki^7^. Malene Johanne Petersen^1^ (ORCID [0000-0002-0531-8531](https://orcid.org/0000-0002-0531-8531)). Heidi Segers^8^ (ORCID [0000-0002-3604-7850](http://orcid.org/0000-0002-3604-7850)). Inge Margriet van der Sluis^9^. Maria Thastrup^1^ (ORCID  0000-0001-9950-5911). Goda Vaitkeviciene^10^. Kjeld Schmiegelow^1,11*^ (ORCID 0000-0002-0829-4993). Linea Natalie Toksvang^1*^ (ORCID 0000-0002-9871-7637).

*Shared last authorship

1 Department of Pediatrics and Adolescent Medicine, University Hospital Rigshospitalet, Copenhagen, Denmark

2 Departamento de Pediatria, Instituto Português de Oncologia Lisboa Francisco Gentil, Lisbon, Portugal.

3 University Medical Center Hamburg-Eppendorf, Hamburg, Germany

4 Astrid Lindgren Children's Hospital, Karolinska University Hospital and Department of Women's and Children's Health, Karolinska Institutet, Stockholm, Sweden

5 Tallinn Children's Hospital, Tallinn, Estonia

6 Dept of Ped hematology and oncology, Oslo University Hospital, Norway.

7 Department of pediatrics, Oulu University Hospital and Research Unit of Clinical medicine, University of Oulu, Oulu, Finland

8 Department of Pediatric Hemato-Oncology - University Hospitals Leuven / Department of Pediatric Oncology - Catholic University Leuven, Leuven, Belgium

9 Princess Máxima Center for Pediatric Oncology, Utrecht, Netherlands

10 Center for Pediatric Oncology and Hematology, Vilnius University, Vilnius, Lithuania

11 Institute of Clinical Medicine, The Faculty of Medicine, University of Copenhagen, Copenhagen, Denmark

**Online Resource 1: Figure S1 of supplementary Material**


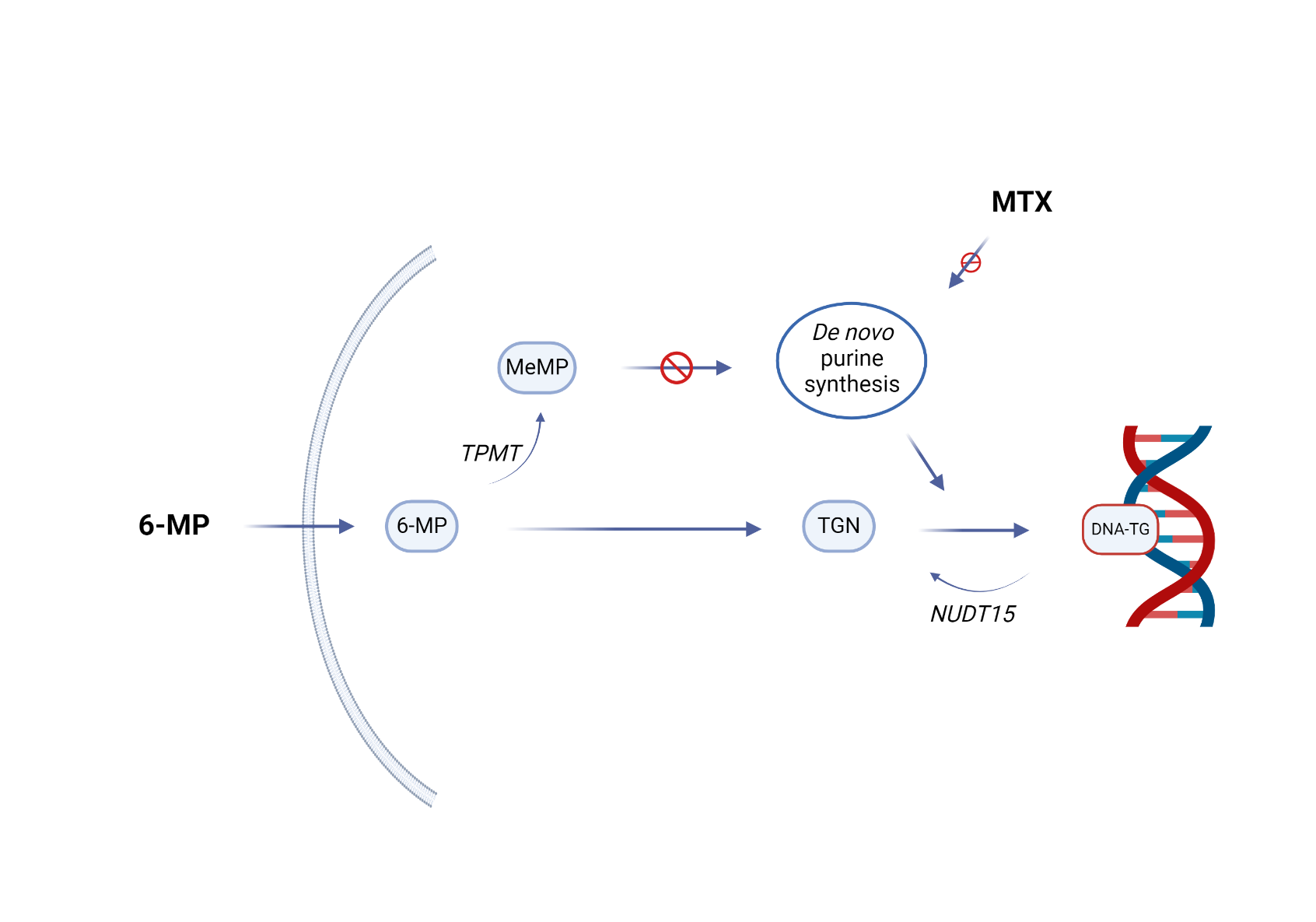


**Fig. S1** Metabolism of 6-mercaptopurine (6-MP). Intercellularly, 6-MP is converted to thioguanine nucleotides (TGN), which are incorporated into DNA (DNA-TG). A fraction of 6-MP and its metabolites are converted to methylated mercaptopurine metabolites (MeMPs) by the enzyme thiopurine S-methyltransferase (TPMT), some of which are inactive, while some inhibit the de novo purine synthesis, reducing the amount of natural guanine and enhancing the formation of DNA-TG. Methotrexate (MTX) also inhibits do novo purine synthesis. Nucleoside diphosphatase, encoded by the nudix hydrolase 15 (NUDT15) gene, counteracts incorporation of TGN into DNA by dephosphorylation of TGNs. Created with BioRender.com

**Online Resource 2: Text S1 of supplementary material. Inclusion and exclusion criteria, directly cited from the AllTogether1 master protocol (EU CT nr 2022-501050-11-01, version 2.2).**

“Inclusion criteria^[[1]](#footnote-1)^:

1. Patients newly diagnosed with T-lymphoblastic (T-cell) or B-lymphoblastic precursor (BCP) leukaemia (ALL) according to the WHO-classification of Tumours of Haematopoietic and Lymphoid Tissues (Revised 4th edition 2017) and with a diagnosis confirmed by an accredited laboratory at a participating paediatric oncology or adult haematology centre.

2. Age ≥ 365 days and < 46 years (one day before 46th birthday) at the time of diagnosis.

3. Informed consent signed by the patient and/or parents/legal guardians according to country-specific age-related guidelines (*http://www.ema.europa.eu/docs/en_GB/document_library/Other/2015/12/WC500199234.pdf* ).

4. The ALL diagnosis should be confirmed by an accredited laboratory at a participating paediatric oncology or adult haematology centre.

5. The patient should be diagnosed and treated at a participating paediatric oncology or adult haematology centre in the participating countries.

6. The patient should be a resident in one of the participating countries on a permanent basis or should intend to settle in a participating country, for instance by an application for asylum. Patients who are visiting the country as tourists should not be included. However, returning expatriots and patients who intend to stay at least for the duration of the treatment with primary diagnosis abroad may be included if no treatment has been administered and the diagnostic procedures are repeated at a participating centre.

7. All women of childbearing potential (WOCBP) have to have a negative pregnancy test within 2 weeks prior to the start of treatment.

8. For each intervention/randomisation an additional set of inclusion-criteria is provided.

Exclusion criteria

1. Age < 365 days at diagnosis (infant ALL) or >45 years at diagnosis.

2. Patients with a previous malignant diagnosis (ALL as a second malignant neoplasm - SMN).

3. Relapse of ALL.

4. Patients with mature B-ALL (as defined by Surface Ig positivity or documented presence of one of the t(8;14), t(2;8), t(8;22) translocations and breakpoint as in BALL).

5. Patients with Ph-positive ALL (documented presence of t(9;22)(q34;q11) and/or of the *BCR*-*ABL1* fusion transcript). These patients will be transferred to an adequate trial for t(9;22) if available.

6. ALL prone syndromes (e.g. Li-Fraumeni syndrome, germline ETV6 mutation), except for Down syndrome. Exploration for such ALL prone syndromes is not mandatory.

7. Treatment with systemic corticosteroids (>10mg/m2/day) for more than one week and/or other chemotherapeutic agents in a 4-week interval prior to diagnosis (pretreatment).

8. Pre-existing contraindications to any treatment according to the ALLTogether ALLTogether1 Protocol Version 2.2 09 Sep 2020 25 protocol (constitutional or acquired disease prior to the diagnosis of ALL preventing adequate treatment).

9. Any other disease or condition, as determined by the investigator, which could interfere with the participation in the study according to the study protocol, or with the ability of the patients to cooperate and comply with the study procedures.

10. Women of childbearing potential who are pregnant at the time of diagnosis.

11. Women of childbearing potential and fertile men who are sexually active and are unwilling to use adequate contraception during therapy. Efficient birth control is required, see section 17.7.

12. Female patients, who are breast-feeding.

13. Essential data missing from the registration of characteristics at diagnosis (in consultation with the protocol chair).”

**Online Resource 3: Figure S2 of supplementary material**

**
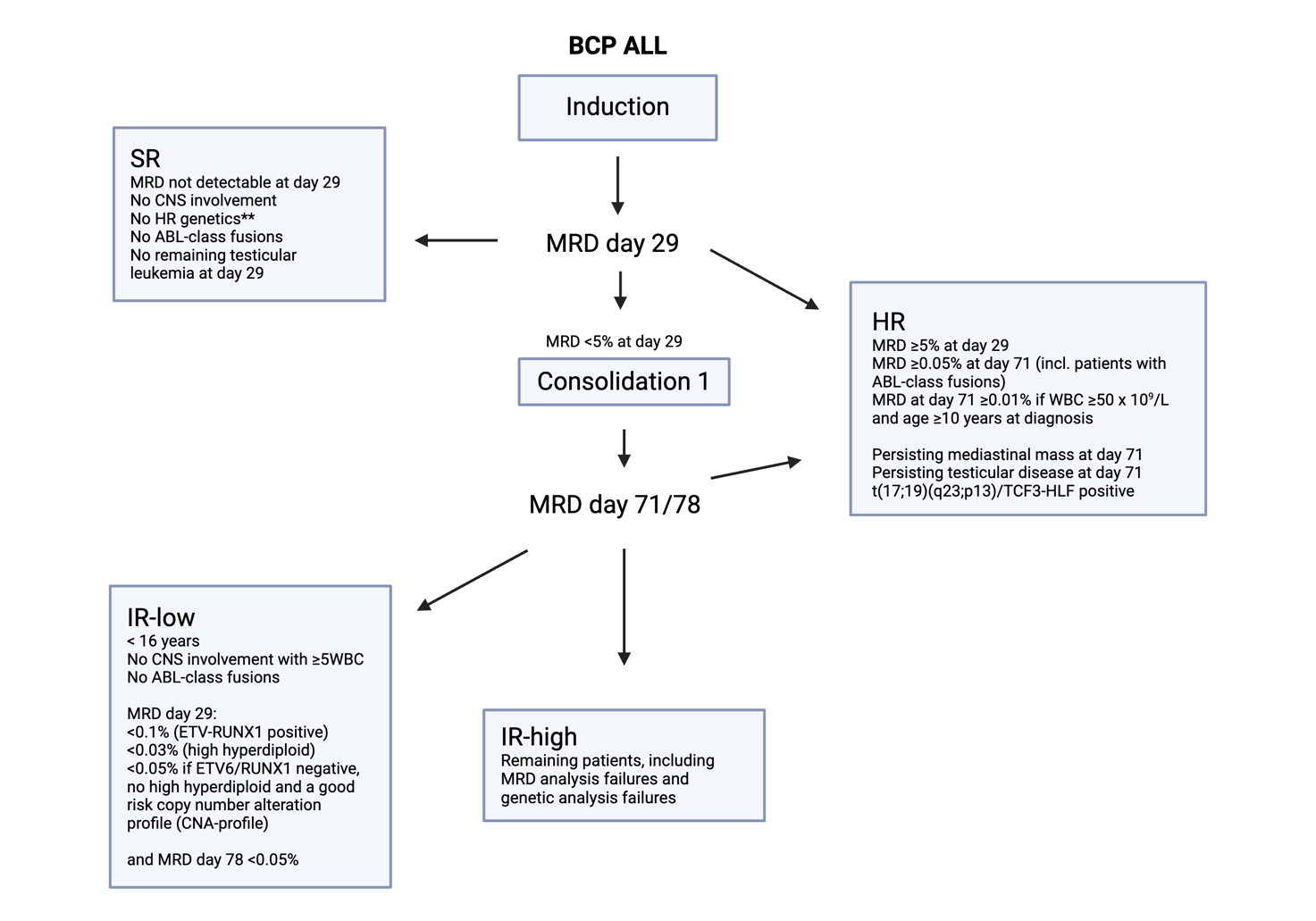
 Fig. S2** Flowchart of the stratification process in the ALLTogether1 (A2G1) treatment protocol for patients with B-cell-precursor (BCP) ALL. SR: standard risk, IR: intermediate risk, HR: high risk, MRD: minimal residual disease. *high risk genetics: KMT2A/MLL gene fusions, near haploidy, low hypodiploidy (<40 chromosomes), iAMP21, t(17;19)/TCF3-HLF. Created with BioRender.com, with inspiration from the AllTogether1 master protocol (EU CT nr 2022-501050-11-01, version 2.2)

**Online resource 4: Figure S3 of supplementary material**

**
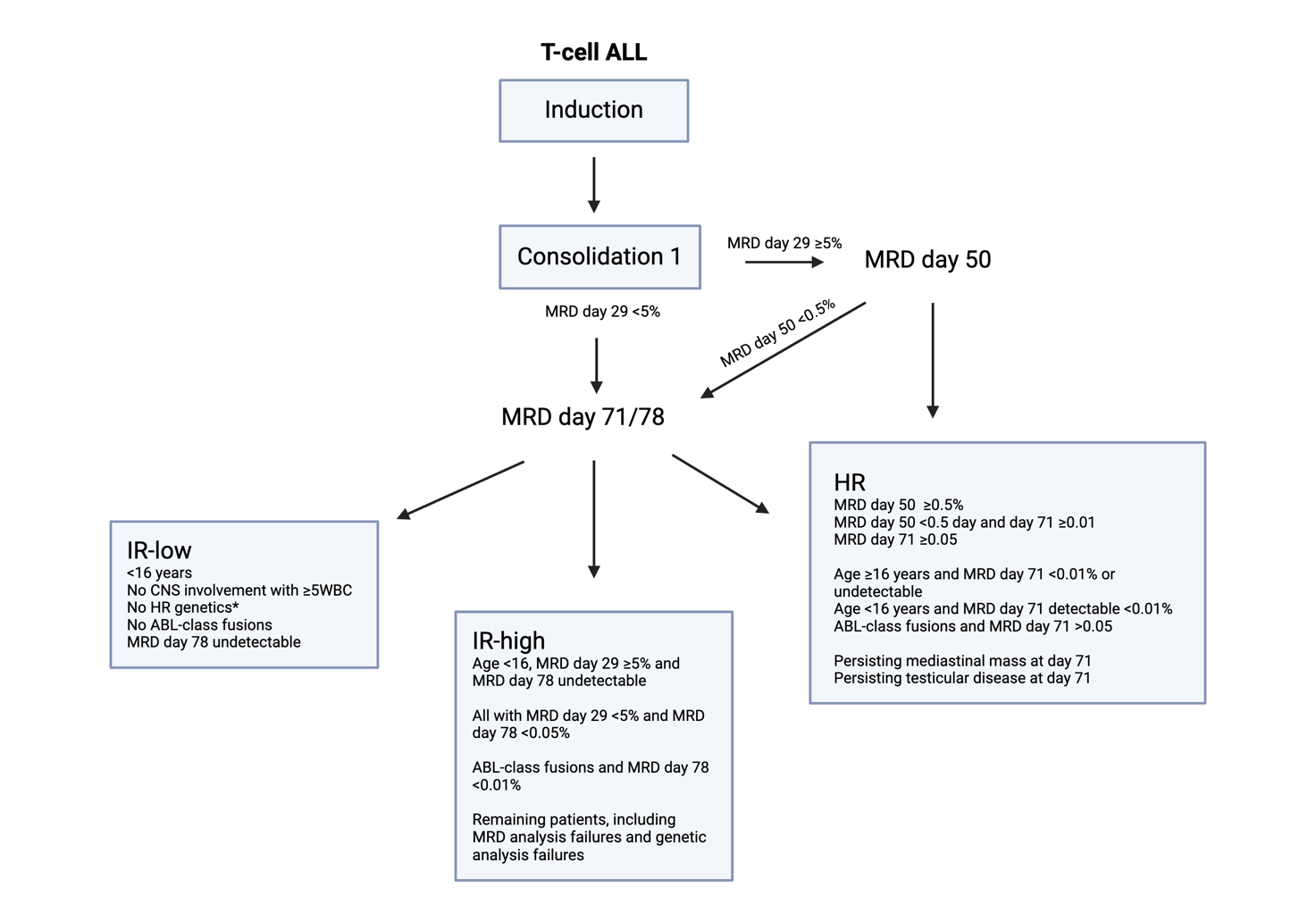
**

**Fig. S3** Flowchart of the stratification process in the ALLTogether1 (A2G1) treatment protocol for patients with T-cell ALL. SR: standard risk, IR: intermediate risk, HR: high risk, MRD: minimal residual disease. *high risk genetics: KMT2A/MLL gene fusions, near haploidy, low hypodiploidy (<40 chromosomes), iAMP21, t(17;19)/TCF3-HLF. Created with BioRender.com, with inspiration from the AllTogether1 master protocol (EU CT nr 2022-501050-11-01, version 2.2)

**Online resource 5: Figure S4 of supplementary material**

**
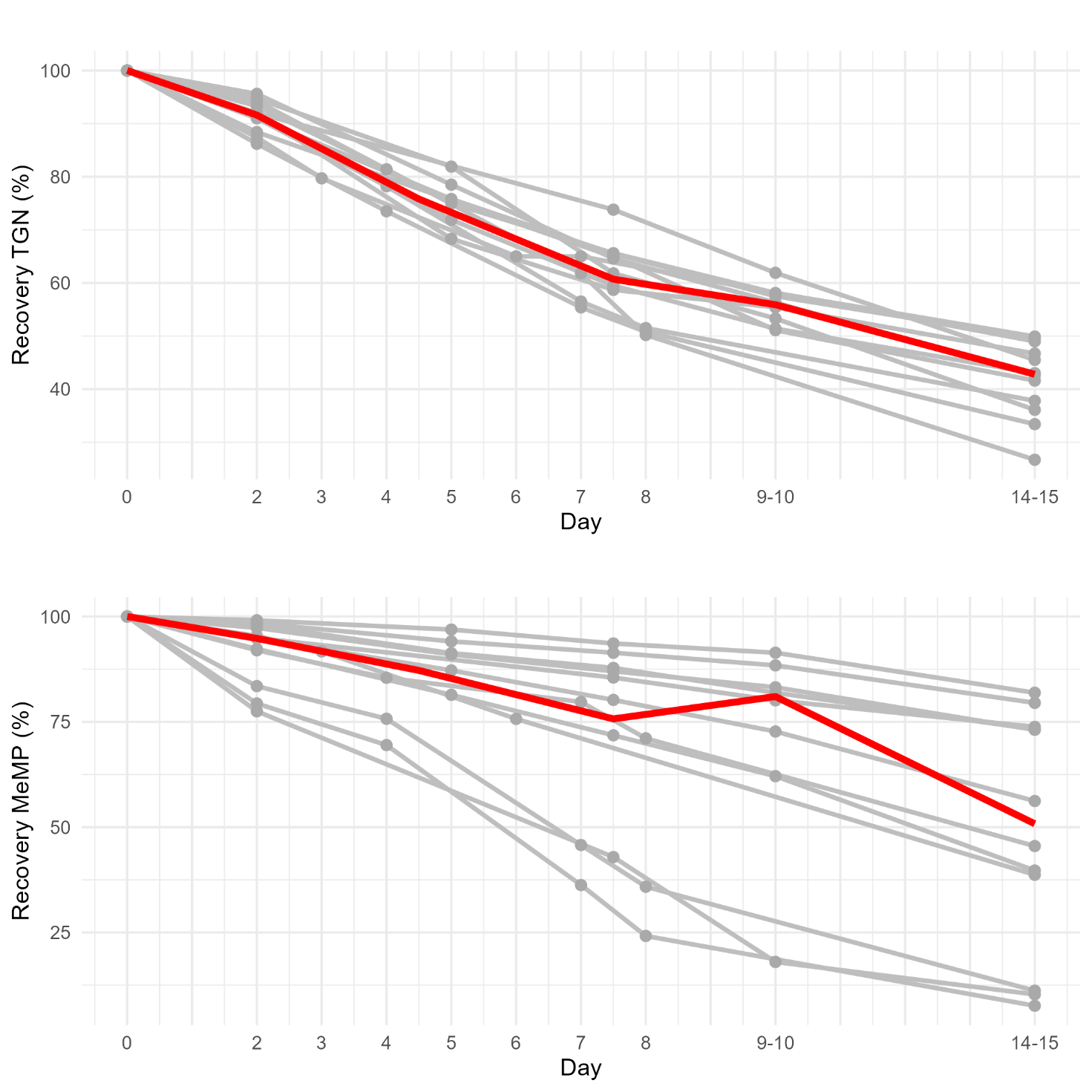
**

**Fig. S4** Plots illustrating the recovery of thioguanine nucleotides (TGN) and methylated mercaptopurine metabolites (MeMP) at room temperature (25℃) during a 14-day period, in samples from 12 patients. Red line: median recovery. Stability analyses were performed at the Peadiatric Oncology Research Laboratory in Copenhagen, Denmark

**Online resource 6: Text S2 of supplementary material**

**Detailed description of statical analyses**

Scatterplots: Pairwise plots of thioguanine nucleotides (TGN), methylated mercaptopurine metabolites (MeMP), DNA incorporated TGN (DNA-TG) and dose of 6-mercaptopurine (6-MP) were visualized using locally estimated scatterplot smoothing (LOESS), for 6-MP metabolites on log-transformed data. The values of metabolites below the detection limit and zero-values of 6-MP dose were not included as x-values in the smoothing. Samples with corresponding missing x-values were not included as y-values in the smoothing, but y-values of metabolites below the detection limit were included as y-values equal to the lowest observed non-zero value of the variable, to reduce the upward bias of the smoothed curve.

Linear mixed effects models and logistic regression models: Associations of DNA-TG with MeMP, TGN and 6-MP dose were assessed on logarithmically transformed data, using linear mixed effects models (R-function *lmer),* and reported as the relative change in DNA-TG per 20% increase of 6-MP dose (mg/m^2^/day), and per doubling of TGN or MeMP. We assessed the TPMT-genotype-specific associations of DNA-TG with the 6-MP dose (mg/m^2^/day). TPMT genotype was grouped into heterozygous and homozygous wild type, excluding one sample from a TPMT deficient patient. Associations between 6-MP metabolites and probability of a prescribed treatment interruption were assessed using mixed effects logistic regression models (R function *glmer*) and reported as the odds ratio (OR) for treatment interruption. To improve the linearity of the associations, MeMP and DNA-TG were logarithmically transformed (log2) while TGN was included linearly, based on an individual assessment of each predictor in the models. Therefore, the associations with MeMP and DNA-TG were reported per doubling, and the associations with TGN were reported per 100 nmol/mmol hemoglobin. In statistical analyses considering MeMP, TGN, and/or 6‑MP dose as predictors, the values below the detection limit and the zero values were set to a constant close to the median of the specific variable; to ensure the analyses were not distorted, we included for each predictor a variable identifying these modified values in the analyses.

All associations were assessed in unadjusted models, mutually adjusted models, a model additionally adjusted for transport time of the samples, and a model further adjusted for age and sex, and the association 6-MP metabolites with probability of a prescribed treatment interruption was further assessed when excluding samples with more than two transport days. Furthermore, all statistical analyses included a random intercept of subject and treatment center to account for the correlation between observations on the same patient or on patients from the same treatment center.

Receiver-operating-characteristic (ROC) curves: Sensitivity and specificity of the logistic regression models were compared graphically by ROC curves, which were made using the predicted probabilities of treatment interruption based on the estimated fixed effects of the mutually adjusted model, and the mutually adjusted model with only DNA-TG and TGN. The area under the curve (AUC) and selected partial areas under the curve (pAUC) were calculated for each ROC curve.

**Online Resource 7: Table S1 of supplementary material**

| **Country** | **No patients**  **(n=368)** | **No samples***  **(n=3,074)** | **Median samples* per patient** | **Median days between samples*** |
| --- | --- | --- | --- | --- |
| Belgium | 76 | 695 | 10 | 28 |
| Denmark | 55 | 798 | 13 | 25.8 |
| Estonia | 5 | 8 | 2 | 64 |
| Finland | 53 | 453 | 8 | 29 |
| Germany | 11 | 21 | 1 | 70.5 |
| Lithuania | 3 | 13 | 3 | 62 |
| Netherlands | 68 | 323 | 5 | 70 |
| Norway | 37 | 351 | 9 | 29 |
| Portugal | 1 | 1 | 1 | - |
| Sweden | 59 | 411 | 5 | 29.5 |

**Table S1** Overview of sample collection across participating countries. *Blood samples analyzed for 6-mercaptopurine metabolites

**Online Resource 8: Table S2 of supplementary material**

|  | | | **All patients**  (n=368) | | **Patients with at least one sample with MeMP, TGN or both below the reporting back limit***  (n=96) | **Patients with at least one sample with both MeMP and TGN below the reporting back limit***  (n=26) |
| --- | --- | --- | --- | --- | --- | --- |
| **Age at diagnosis** (years) | | |  |  |  |  |
|  | | Median [Min, Max] |  | 4.7 [0.4, 17.2] | 4.3 [1.2, 16.7] | 5.0 [1.4, 16.7] |
| **Sex** | | |  |  |  |  |
|  | | Male  Female |  | 195 (53.0%)  173 (47.0%) | 52 (54.2%)  44 (45.8%) | 17 (65.4%)  9 (34.6%) |
| **Phenotype** | | |  |  |  |  |
|  | | B-lymphoblastic precursor ALL  T-lymphoblastic ALL  Mixed phenotype ALL |  | 343 (93.2%)  23 (6.3%)  2 (0.5%) | 85 (88.5%)  9 (9.4%)  2 (2.0%) | 24 (92.3%)  2 (7.7%) |
| **Risk group** | | |  |  |  |  |
|  | | Standard risk  Standard risk Down syndrome  Intermediate risk-low  Intermediate risk-high  Intermediate risk Down syndrome |  | 121 (32.9%)  1 (0.3%)  185 (50.3%)  52 (14.1%)  9 (2.4%) | 22 (22.9%)  1 (1.0%)  51 (53.1%)  21 (21.9%)  1 (1.0%) | 4 (15.4%)    13 (50.0%)  8 (30.8%)  1 (3.8%) |
| **TPMT genotype** | | |  |  |  |  |
|  | | Homozygous wild type  Heterozygous  Homozygous deficient  Not analyzed |  | 333 (90.5%)  22 (6.0%)  1 (0.3%)  12 (3.3%) | 87 (90.6%)  8 (8.3%)  1 (1.0%) | 26 (100%) |
| **NUDT15 genotype** | | |  |  |  |  |
|  | Homozygous wild type  Heterozygous  Not analyzed** | |  | 100 (27.2%)  3 (0.8%)  265 (72%) | 24 (25.0%)  1 (1.0%)  71 (74.0%) | 5 (19.2%)  1 (3.8%)  20 (76.9%) |
| **Metabolite samples per patient** | | |  |  |  |  |
|  | | Median [Min, Max] |  | 7 [1, 34] | 11.5 [1, 34] | 10 [1, 27] |
| **Median days between metabolite samples per patient** | | |  |  |  |  |
|  | | Median [Min, Max] |  | 29 [12.5, 209] | 28 [13, 98] | 28 [14, 84.5] |

**Table S2** Patient characteristics. 6-MP: 6-mercaptopurine, TPMT: thiopurine S-methyltransferase. NUDT15: nudix hydrolase 15. *Limits of thioguanine nucleotides (TGN) or methylated mercaptopurine metabolites (MeMP) in erythrocytes for generation of a reporting back notice to the treating physician in the ALLTogether (A2G) Maintenance Therapy sub-study. **NUDT15 analyses only mandatory for patients of Asian ancestry in the A2G1 protocol.

**Online Resource 9: Figure S5 of supplementary material**


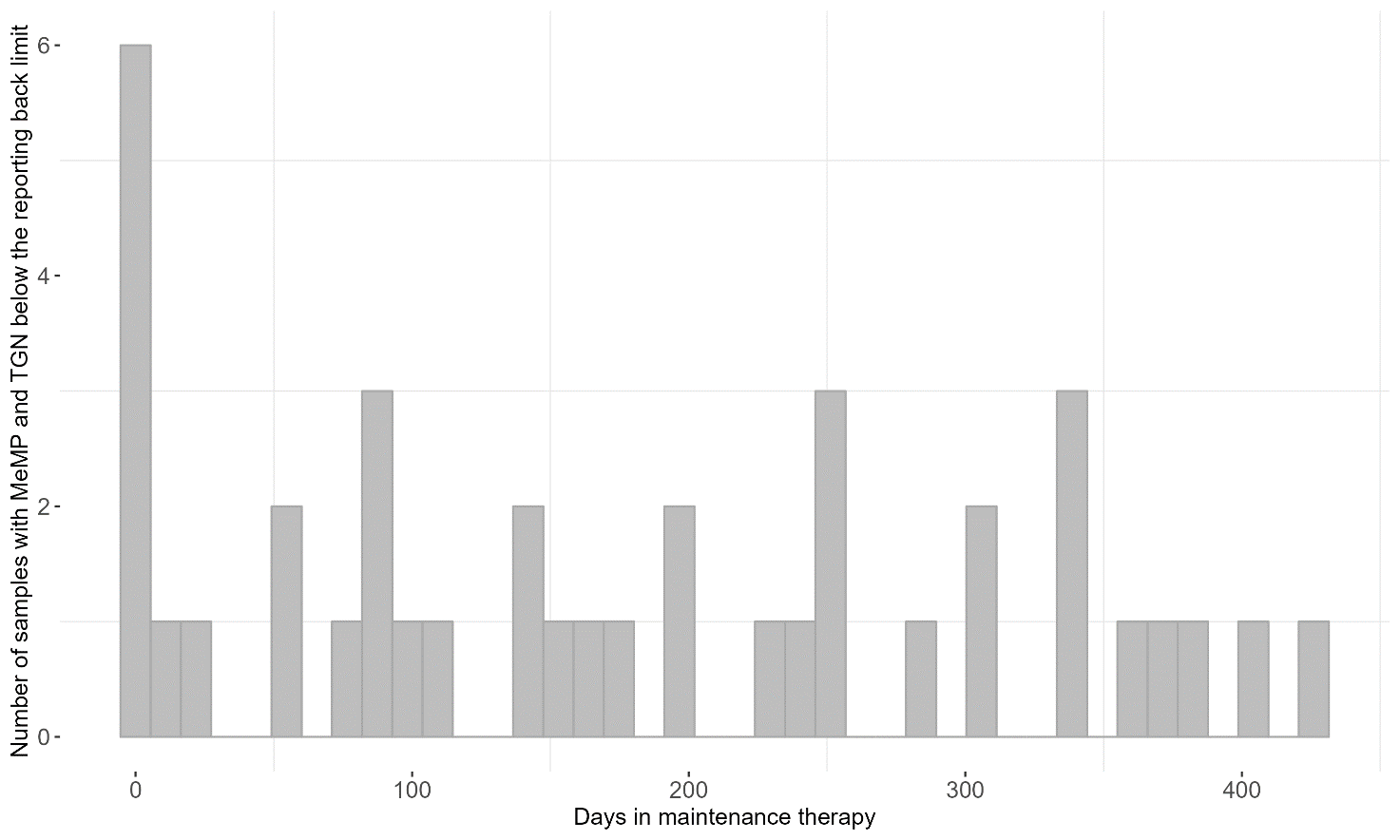


**Fig. S5** Histogram of timepoints (days in maintenance therapy) for collection of blood samples with both thioguanine nucleotides (TGN) and methylated mercaptopurine metabolites (MeMP) below the reporting back limits in the ALLTogether Maintenance Therapy sub-study.

**Online Resource 10: Table S3 of supplementary material**

|  | **_m_DNA-TG** | | | **_m_MeMP** | | | **_m_TGN** | | |
| --- | --- | --- | --- | --- | --- | --- | --- | --- | --- |
| **Sex (n)** |  |  |  |  |  |  |  |  |  |
| Males (196), median | 638 | | | 4825 | | | 226 | | |
| Females (173), median | 512 | | | 4508 | | | 208 | | |
| p-value (males, females) | **0.0028** | | | 0.4540 | | | 0.3053 | | |
|  |  |  |  |  |  |  |  |  |  |
| **Risk group (n)** |  |  |  |  |  |  |  |  |  |
| SR (121), median | 659 | | | 5185 | | | 219 | | |
| IR-low (185), median | 541 | | | 4784 | | | 212 | | |
| IR-high (52), median | 559 | | | 3997 | | | 233 | | |
| p-value (SR, IR-low) | **0.0053** | | | 0.6151 | | | 0.2539 | | |
| p-value (SR, IR-high) | **0.04717** | | | 0.0753 | | | 0.9164 | | |
| p-value (IR-low, IR-high) | 0.8459 | | | 0.0837 | | | 0.4504 | | |
|  |  | | |  | | |  | | |
| **ALL phenotype (n)** |  |  |  |  |  |  |  |  |  |
| Pre-B-cell (343), median | 580 | | | 4706 | | | 217 | | |
| T-cell (23), median | 602 | | | 5956 | | | 231 | | |
| p-value (Pre-B-cell, T-cell) | 0.9715 | | | 0.7998 | | | 0.9705 | | |

**Table S3** Median values of DNA incorporated thioguanine nucleotides (DNA-TG), methylated mercaptopurine metabolites (MeMP) and thioguanine nucleotides (TGN) in different groups of patients. Groups with less than 10 patients are not included. P-values are from Mann Whitney U tests, comparing 6-MP metabolites across the different groups using median DNA-TG/MeMP/TGN for each patient (_m_DNA-TG/_m_MeMP/_m_TGN). SR: standard risk, IR-low: intermediate risk low, IR-high: intermediate risk high.

**Online Resource 11: Figure S6 of supplementary material**

**
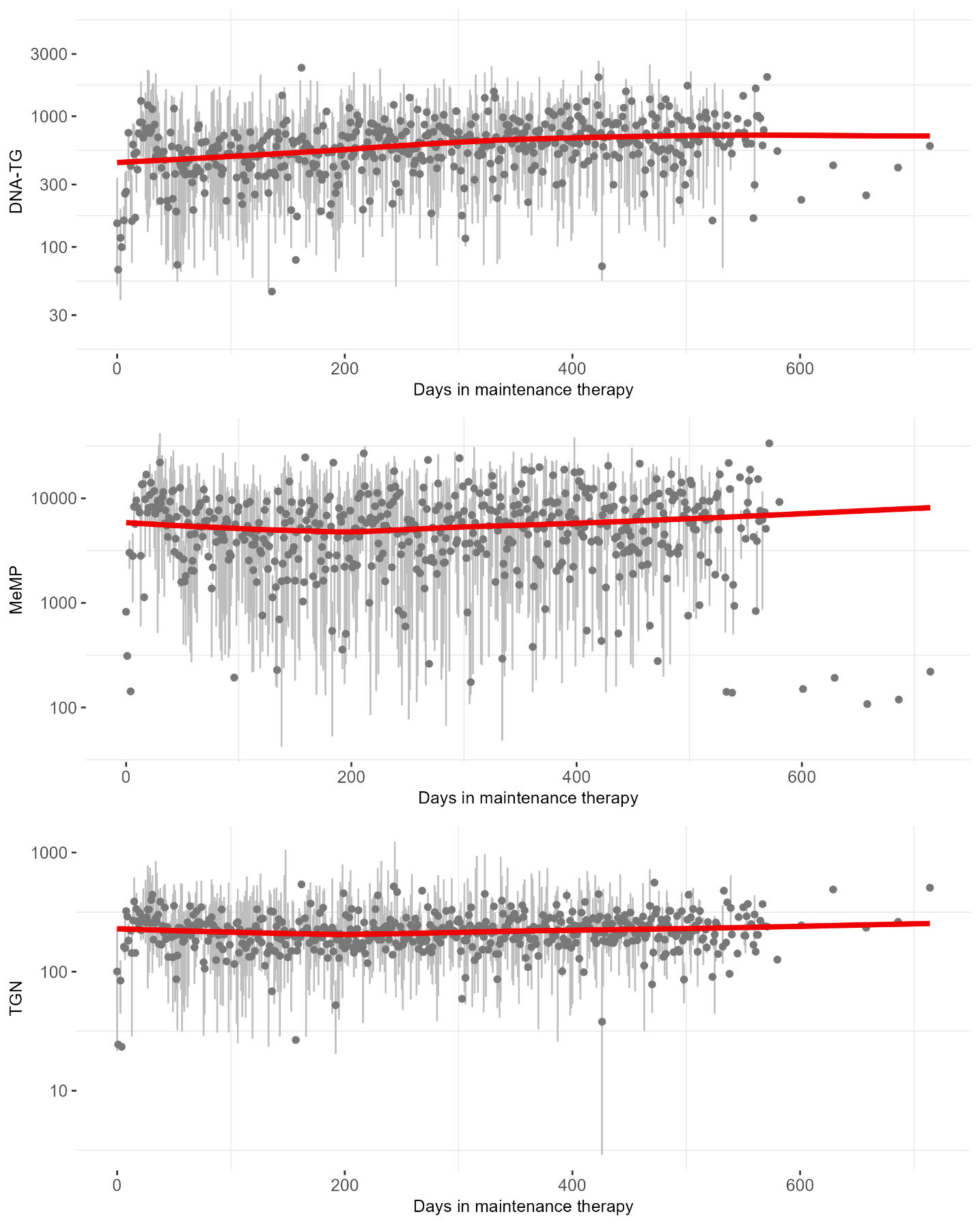
Fig. S6** Plot of the median total DNA incorporated thioguanine nucleotides (DNA-TG), total methylated mercaptopurine metabolites (MeMP), and total thioguanine nucleotides (TGN) over time in maintenance therapy, with a locally estimated scatterplot smoothing (red line). The median is illustrated for each day in maintenance therapy (points), along with the 95^th^ percentile for each day (vertical lines)

**Online Resource 12: Figure S7 of supplementary material**


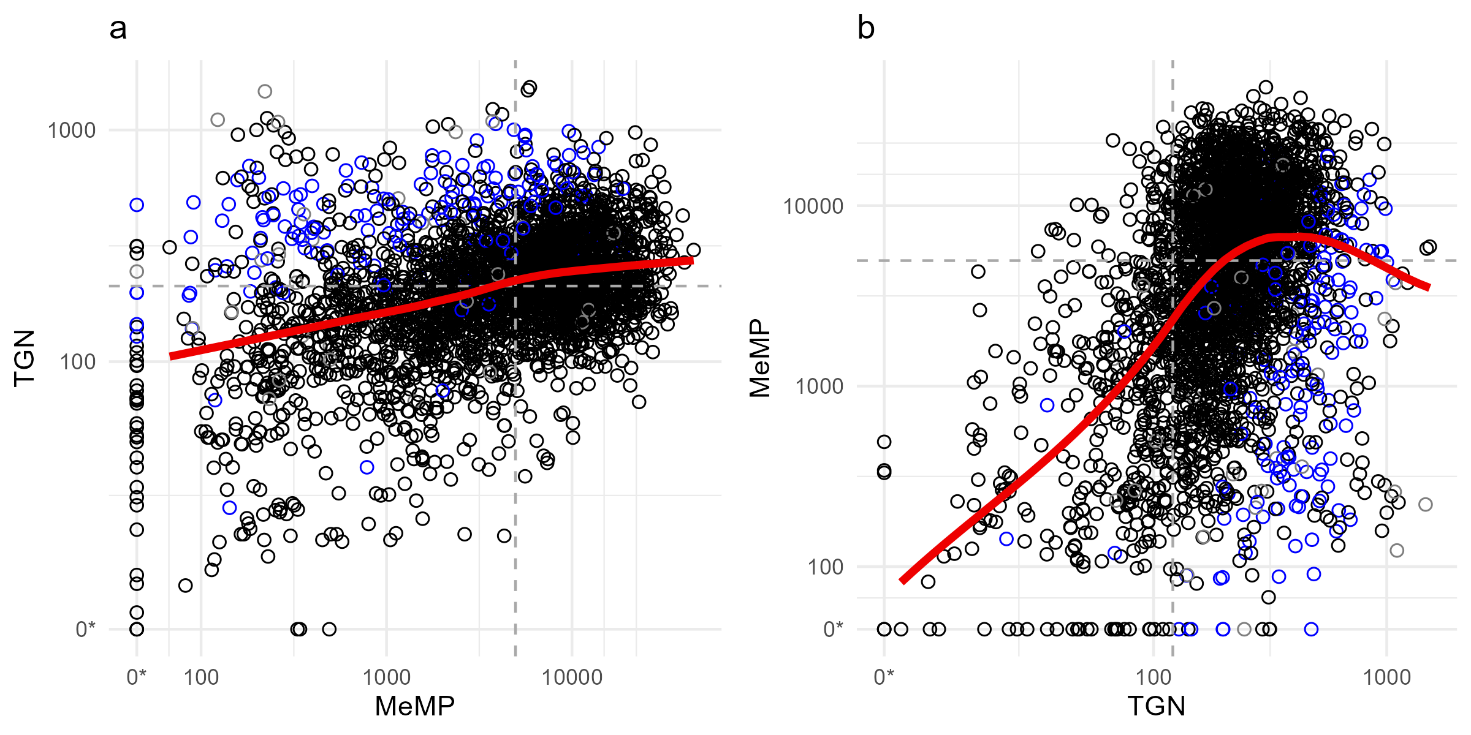


**Fig. S7** Scatterplots of association between thioguanine nucleotides (TGN) and methylated mercaptopurine metabolites (MeMP), with locally estimated scatterplot smoothing (red line). Black points: samples from one thiopurine S-methyltransferase (TPMT) homozygous wild type patients, blue points: samples from TPMT heterozygous patients. Dotted line: median value. *Below the detection limit

**Online Resource 13: Table S4 of supplementary material**

|  | **Mutually adjusted model** | | | **Additionally adjusted for time of sample transport** | | | **Additionally adjusted for transport time, age, and sex** | | |
| --- | --- | --- | --- | --- | --- | --- | --- | --- | --- |
|  | Estimate | 95% CI | p-value | Estimate | 95% CI | p-value | Estimate | 95% CI | p-value |
| MeMP per doubling | 1.101 | 1.085-1.116 | <0.0001 | 1.100 | 1.085-1.116 | <0.0001 | 1.099 | 1.084-1.115 | <0.0001 |
| TGN per doubling | 1.723 | 1.675-1.773 | <0.0001 | 1.746 | 1.697-1.796 | <0.0001 | 1.747 | 1.698-1.796 | <0.0001 |

**Table S4** Associations of DNA incorporated thioguanine (DNA-TG) with thioguanine nucleotides (TGN) and methylated mercaptopurine metabolites (MeMP) in linear mixed effects models, with a random effect of patient and treatment center. Analyses were performed on complete datasets of included variables (mutually adjusted model: 2,990 samples from 364 patients, additionally adjusted for transport time: 2,984 samples from 364 patients, additionally adjusted for transport time, age and sex: 2,980 samples from 363 patients)

**Online Resource 14: Table S5 of supplementary material**

|  | **Mutually adjusted model** | | | **Additionally adjusted for transport time** | | | **Additionally adjusted for transport time, age, and sex** | | |
| --- | --- | --- | --- | --- | --- | --- | --- | --- | --- |
|  | Estimate | 95% CI | p-value | Estimate | 95% CI | p-value | Estimate | 95% CI | p-value |
| Dose 6-MP per 20% increase for TPMT homozygous wild type patients (n=317) | 1.083 | 1.073-1.093 | <0.0001 | 1.083 | 1.073-1.094 | <0.0001 | 1.083 | 1.073-1.094 | <0.0001 |
| Dose 6-MP per 20% increase for TPMT heterozygous patients (n=21) | 1.132 | 1.086-1.180 | <0.0001 | 1.132 | 1.086-1.180 | <0.0001 | 1.599 | 1.131-1.179 | <0.0001 |

**Table S5** Association of DNA incorporated thioguanine (DNA-TG) with prescribed dose of 6-mercaptopurine (6-MP) in mg/m^2^/day in linear mixed effects models including interaction with thiopurine S-methyltransferase (TPMT) genotype, with a random effect of patient and treatment center. Analyses were performed on complete datasets of included variables (mutually adjusted model: 2,194 samples from 338 patients, additionally adjusted for transport time: 2,191 samples from 338 patients, additionally adjusted for transport time, age and sex: 2,191 samples from 338 patients)

**Online Resource 15: Table S6 of supplementary material**

|  | **Mutually adjusted model** | | | **Additionally adjusted for transport time** | | | **Additionally adjusted for transport time, age, and sex** | | | **Mutually adjusted model, excluding samples with >2 days of transport** | | |
| --- | --- | --- | --- | --- | --- | --- | --- | --- | --- | --- | --- | --- |
|  | OR | 95% CI | p-value | OR | 95% CI | p-value | OR | 95% CI | p-value | OR | 95% CI | p-value |
| DNA-TG per doubling | 0.41 | 0.29-0.58 | <<0.0001 | 0.43 | 0.30-0.59 | <<0.0001 | 0.43 | 0.31-0.60 | <<0.0001 | 0.35 | 0.36-0.77 | 0.001 |
| TGN per 100 nmol/mmol hemoglobin | 0.13 | 0.06-0.26 | <<0.0001 | 0.11 | 0.05-0.24 | <<0.0001 | 0.11 | 0.06-0.24 | <<0.0001 | 0.11 | 0.05-0.25 | <<0.0001 |
| MeMP per doubling | 1.34 | 1.07-1.68 | 0.011 | 1.33 | 1.06-1.66 | 0.014 | 1.33 | 1.07-1.67 | 0.012 | 1.10 | 0.84-1.45 | 0.485 |

**Table S6** Association of DNA incorporated thioguanine (DNA-TG) with probability of a prescribed treatment interruption in mixed effects logistic regression models, with a random effect of patient and treatment center. Analyses were performed on a complete dataset of the included variables (2,455 samples from 349 patients). TGN: thioguanine nucleotides, MeMP: methylated mercaptopurine metabolites, OR: odds ratio

1. Criteria is expanded to allow inclusion of infants without KMT2A mutations. [↑](#footnote-ref-1)
